# Supplementary material for: Analysis of White Mulberry Leaves and Dietary Supplements, ATR-FTIR Combined with Chemometrics for the Rapid Determination of 1-Deoxynojirimycin
Source: Nutrients. 2022 Dec 10;14(24):5276. doi: 10.3390/nu14245276 (PMC9781008; doi:10.3390/nu14245276)
Supplement: Supplementary file 1 [file nutrients-14-05276-s001.zip › nutrients-2077885-supplementary.pdf]

Supplementary material

Analysis of White Mulberry leaves and dietary supplements. ATR-FTIR combined with chemometrics for the rapid determination of 1-deoxynojirimycin.

Agata Walkowiak-Bródka [1], Natalia Piekuś-Słomka [1], Kacper Wnuk [2] and Bogumiła Kupcewicz [1,*]

[1] Department of Inorganic and Analytical Chemistry, Faculty of Pharmacy, Nicolaus Copernicus University in Torun; kizchemanal@cm.umk.pl

[2] Department of Biostatistics and Biomedical Systems Theory, Faculty of Pharmacy, Nicolaus Copernicus University in Torun; biostat@cm.umk.pl

*Correspondence: kupcewicz@cm.umk.pl

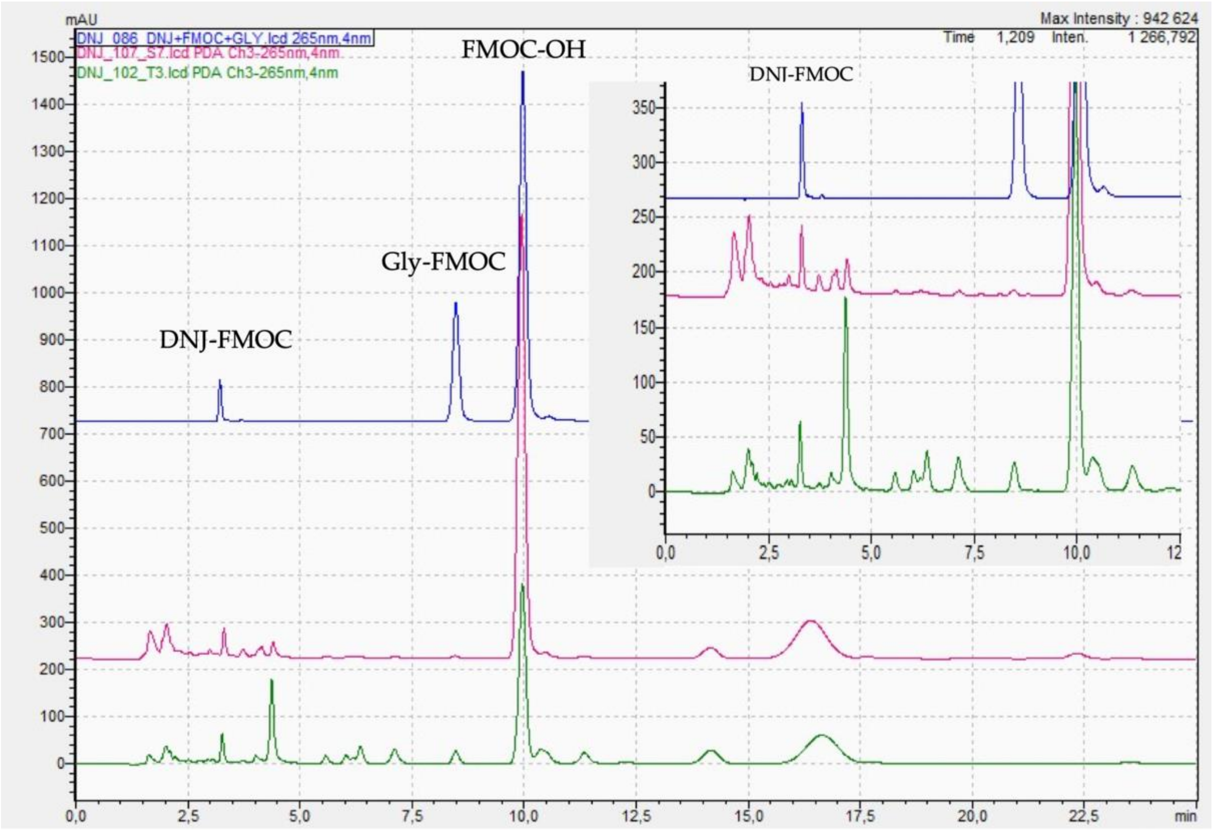

Figure S1. Chromatograms of derivatized standard – DNJ (blue line) and exemplary derivatized samples: extract from a dietary supplement (pink line) and from dry Mulberry leaves (green line).

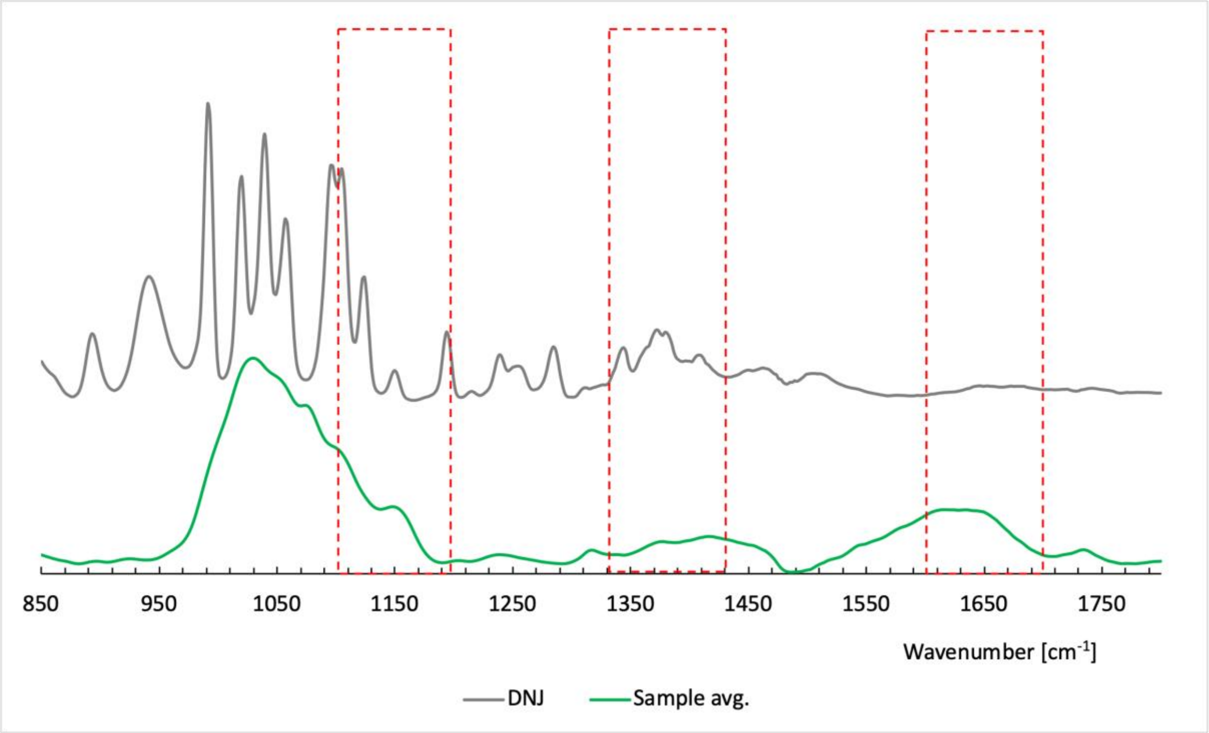

Figure S2. The average spectrum of tested samples: dietary supplements and teas (green line) and the DNJ spectrum (grey line). Spectra were preprocessed by SNV. The red boxes indicate spectral intervals used to build the iPLS model for DNJ prediction.
